# *In vitro* activity of mecillinam, temocillin and nitroxoline against MDR Enterobacterales

**DOI:** 10.1093/jacamr/dlac059

**Published:** 2022-06-16

**Authors:** Lars Plambeck, Frieder Fuchs, Janko Sattler, Axel Hamprecht

**Affiliations:** Institute for Medical Microbiology, Immunology and Hygiene, University of Cologne, Medical faculty and University Hospital of Cologne, Cologne, Germany; Institute for Medical Microbiology, Immunology and Hygiene, University of Cologne, Medical faculty and University Hospital of Cologne, Cologne, Germany; Institute for Medical Microbiology, Immunology and Hygiene, University of Cologne, Medical faculty and University Hospital of Cologne, Cologne, Germany; Institute for Medical Microbiology, Immunology and Hygiene, University of Cologne, Medical faculty and University Hospital of Cologne, Cologne, Germany; German Centre for Infection Research, partner site Bonn-Cologne (DZIF), Cologne, Germany; Institute for Medical Microbiology and Virology, University of Oldenburg, Oldenburg, Germany

## Abstract

**Background:**

With increasing resistance to common antibiotics the treatment of urinary tract infections has become challenging and alternative therapeutic options are needed. In the present study, we evaluate the activity of three older and less frequently used antibiotics against MDR Enterobacterales.

**Methods:**

Susceptibility of mecillinam, temocillin and nitroxoline was assessed in Enterobacterales isolated from urinary specimens with elevated MICs of third-generation cephalosporins. Susceptibility was determined by the recommended reference MIC methods and additionally by disc diffusion. All isolates were characterized for common β-lactamases by phenotypic and molecular assays.

**Results:**

In total 394 Enterobacterales were included. The most common resistance mechanisms were ESBLs (*n *= 273), AmpC (*n *= 132), carbapenemases [*n *= 12, including OXA-48-like (*n *= 8), VIM (*n *= 2), KPC (*n *= 1) and NDM (*n *= 1)] or others (*n *= 2). Resistance was observed in 59% of isolates to ceftazidime, in 41% to piperacillin/tazobactam and in 54% to ciprofloxacin. In comparison, resistance was less frequent against mecillinam (15%), temocillin (13%) or nitroxoline (2%). Mecillinam showed higher activity in *Enterobacter* spp., *Escherichia coli* and in OXA-48-like-producing isolates compared with temocillin, which was more active in *Proteus mirabilis* and in ESBL-producing isolates. Activity of nitroxoline was high against all isolates, including carbapenemase-producing isolates. Correlation between disc diffusion and MIC methods was good for mecillinam and moderate for temocillin and nitroxoline.

**Conclusions:**

Mecillinam, temocillin and nitroxoline show good to excellent *in vitro* activity in MDR Enterobacterales. The activity of mecillinam and temocillin was higher in certain species and restricted depending on β-lactamase production while nitroxoline showed universally high activity irrespective of species or β-lactamase present.

## Introduction

With increasing antibiotic resistance the therapy of urinary tract infections (UTI) has become more and more difficult and the need for alternative therapies has led to regained interest in older drugs such as mecillinam, temocillin and nitroxoline.^1–3^ Gram-negative bacteria resistant to carbapenems or third-generation cephalosporins are particularly concerning.^4^ Facing the paucity in the development of new antibiotics, the revival of older antibiotics has become an important strategy to fight the antimicrobial resistance (AMR) crisis.^5^

Mecillinam, temocillin and nitroxoline were developed in the 1950s–80s.^6–8^ The current use of these drugs is mostly limited to infections of the urinary tract. Pivmecillinam, the oral prodrug of mecillinam, has been used in Scandinavian countries for the treatment of uncomplicated UTI (uUTI) for decades. Both pivmecillinam and nitroxoline are recommended as oral agents in the guideline on uUTI in Germany.^9^ In contrast parenteral temocillin is also used for invasive infections, yet experience is limited to some European countries, especially UK, France and Belgium.^10^ All three agents may remain active against MDR pathogens and side effects are rare.^2,11–16^

Mecillinam and temocillin are both β-lactam antibiotics.^2,3^ Nitroxoline is a quinoline derivate and the mode of action is based on ion chelation with subsequent effects on microbial enzymatic pathways (including transcription factors) and effects on the charge of cellular compartments.^2^ Mecillinam, temocillin and nitroxoline differ in the route of administration: For mecillinam oral and IV administration is possible; in contrast temocillin can only be applied IV while nitroxoline is available only in oral form.

With the increasing number of infections by MDR strains there is regained interest in these older antibiotics.^17^ Hence, over the past 12 years, EUCAST has issued breakpoints for all three drugs, which are currently limited to certain species. Additionally, breakpoints for mecillinam and nitroxoline apply only to uUTI while for temocillin breakpoints are also valid for complicated UTI (cUTI).^18^ To date, only limited data on susceptibility testing of these drugs and correlation of testing methods are available, as reference methodologies are laborious (i.e. agar dilution for mecillinam) and not available in most clinical microbiology laboratories.

Currently only few studies on the susceptibility of mecillinam, temocillin and nitroxoline have been performed and to the best of our knowledge none has compared the activity of all three substances in MDR Enterobacterales isolates.

The aim of the present study was therefore to determine the activity of all three drugs in a collection of different MDR uropathogenic Enterobacterales. Secondly, we assessed disc diffusion testing as an alternative method for susceptibility testing of the three drugs and compared it with current reference methods.

## Materials and methods

Enterobacterales with elevated MICs of cefotaxime and/or ceftazidime and/or ertapenem, imipenem or meropenem that had been isolated from urine specimens at the Institute for medical Microbiology of the University Hospital Cologne between March 2019 and January 2020 were included in the study. Susceptibility testing of standard antibiotics was done on a Vitek 2 system using the AST N195 card (bioMérieux, Nürtingen, Germany) and results were interpreted according to EUCAST breakpoints. Isolates were further characterized for ESBL production by the CLSI combination disc test (MAST-group, Bootle, UK) and for AmpC using the cefoxitin/cloxacillin and cefotaxime/cloxacillin disc test (Liofilchem, Roseto degli Abruzzi, Italy), as described previously.^19,20^ In case of elevated carbapenem MICs isolates were characterized by a rapid PCR assay (GeneXpert, Carba-R kit, Cepheid, Sunnyvale, CA, USA), followed by PCR and Sanger sequencing or WGS as described previously.^21–23^ MICs of mecillinam were assessed by agar dilution using mecillinam powder (Molekula, Munich, Germany). MICs of temocillin and nitroxoline were determined by broth microdilution using temocillin powder (Eumedica, Basel, Switzerland) and nitroxoline powder (Rosen Pharma, St. Ingbert, Germany) in 96-well plates. For the three drugs breakpoints defined by EUCAST for selected species were used for all Enterobacterales species to allow comparison of susceptibility rates.

Additionally, susceptibility testing by disc diffusion was performed using 10 μg mecillinam discs (Oxoid, Wesel, Germany), 30 μg nitroxoline discs (Oxoid) and 30 μg temocillin discs (MAST). All susceptibility tests were performed from the same bacterial suspension. *Escherichia coli* ATCC 25922 served as quality control. Spearman’s correlation coefficient for ranked data served to assess correlation of MICs and inhibition zones.

### Ethics

The study was conducted in accordance with the Declaration of Helsinki. All bacterial strains were isolated as part of routine microbiological diagnostics. The requirement for written informed consent was waived due to the observational, retrospective nature of this study.

## Results

In total 394 Enterobacterales (Table 1 and Table S1, available as Supplementary data at *JAC-AMR* Online) were included in the study. *E. coli* was the most common species (*n *= 198), followed by *Klebsiella pneumoniae* (*n *= 66), *Enterobacter* spp. (*n *= 52) and other species (*n *= 78) (Figure S1). The most frequent isolation source was voided midstream urine (245 isolates), catheter urine (*n *= 98) and other sources (*n *= 51). Most patients were female (214/394, 54%), the median age was 66 years. The majority of isolates was cultured from samples of inpatients (258/394, 65%) and from the urological department (137/394, 35%). For 20 of 394 isolates (5%) a coincident bloodstream infection with the same species and resistance phenotype was detected.

**Table 1.** MICs of isolates of mecillinam, temocillin and nitroxoline, stratified by species

Species (*n*)	Mecillinam (S ≤ 8/R > 8 mg/L^a^)			Temocillin (S ≤ 0.001/R > 16 mg/L^a^)			Nitroxoline (S ≤ 16/R > 16 mg/L^a^)		
	MIC_50_ (mg/L)	MIC range (mg/L)	Resistant isolates, *n* (%)	MIC_50_ (mg/L)	MIC range (mg/L)	Resistant isolates, *n* (%)	MIC_50_ (mg/L)	MIC range (mg/L)	Resistant isolates, *n* (%)
*E. coli* (198)	2	0.25 to 64	6 (3)	4	0.5 to >128	8 (4)	2	0.25 to 32	2 (1)
*Klebsiella* spp. (87)	8	0.5 to >128	27 (31)	4	0.5 to >128	13 (15)	4	1 to 64	2 (2)
* K. pneumoniae* (66)	4	0.5 to >128	16 (24)	4	0.5 to >128	8 (12)	4	1 to 64	2 (3)
* K. aerogenes* (12)	2	1 to >128	2 (17)	8	2 to 128	4 (33)	4	1 to 8	0
* K. oxytoca* (9)	64	32 to >128	9 (100)	4	1 to 32	1 (11)	4	1 to 8	0
*Enterobacter* spp. (52)	1	0.125 to >128	2 (4)	8	0.5 to 128	16 (31)	8	0.5 to 64	2 (4)
*C. freundii* (33)	2	0.125 to >128	9 (27)	8	0.25 to >128	11 (33)	4	0.5 to 16	0
*Citrobacter koseri* (1)		8	0		2	0		4	0
*M. morganii* (11)	>128	128 to >128	11 (100)	16	4 to 64	4 (36)	4	0.5 to 16	0
*P. mirabilis* (8)	>128	2 to >128	5 (63)	2	1 to 4	0	4	1 to 8	0
*H. alvei* (3)	2	0.5 to 4	0	8	4 to 8	0	2	2	0
*Raoultella ornithinolytica* (1)		2	0		8	0		8	0
All isolates (394)	2	0.125 to >128	60 (15)	4	0.25 to >128	52 (13)	4	0.25 to 64	6 (2)

S, susceptible; R, resistant.

a Breakpoint according to EUCAST for selected species.

Of all isolates, 273 (69%) tested positive for ESBL production and 132 (34%) showed the phenotype of a derepressed AmpC β-lactamase. One *Klebsiella oxytoca* isolate (0.3%) hyperproduced the chromosomal K1 β-lactamase. Carbapenemases were detected in 12 isolates (3%), including OXA-48-like (*n *= 8), VIM (*n *= 2), KPC (*n *= 1) and NDM (*n *= 1) (Table 2 and Table S1).

**Table 2.** MICs of isolates, stratified by β-lactamases

β-Lactamase (*n*)	Mecillinam (S ≤ 8/R > 8 mg/L^a^)			Temocillin (S ≤ 0.001/R > 16 mg/L^a^)			Nitroxoline (S ≤ 16/R > 16 mg/L^a^)		
	MIC_50/90_ (mg/L)	MIC range (mg/L)	Resistant isolates, *n* (%)	MIC_50/90_ (mg/L)	MIC range (mg/L)	Resistant isolates, *n* (%)	MIC_50/90_ (mg/L)	MIC range (mg/L)	Resistant isolates, *n* (%)
ESBL (273)	4/32	0.25 to >128	34 (12)	4/16	0.5 to >128	20 (7)	4/8	0.25 to 64	4 (1)
AmpC (132)	2/>128	0.125 to >128	25 (19)	8/64	0.25 to >128	36 (27)	4/16	0.5 to 64	2 (2)
ESBL + AmpC (16)	8/>128	1 to >128	4 (25)	4/64	1 to 64	5 (31)	8/16	2 to 16	0
HyperK1 (1)	>128		1 (100)	8		0	4		0
Carbapenemases (12)	64/>128	1 to >128	8 (67)	128/>128	2 to >128	9 (75)	4/8	0.5 to 8	0
KPC (1)	>128		1 (100)	4		0	0.5		0
OXA-48-like (8)	8/64	1 to 128	4 (50)	128/>128	64 to >128	8 (100)	2/8	1 to 8	0
MBL (3) (VIM, NDM)	>128/>128	>128	3 (100)	4/>128	2 to >128	1 (33)	4/8	4 to 8	0

S, susceptible; R, resistant.

a Breakpoint according to EUCAST for selected species.

**Figure 1.**
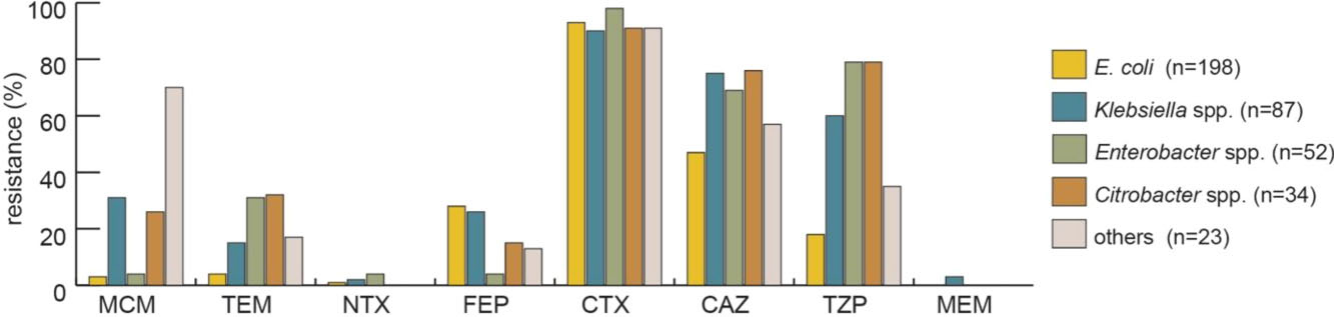
Resistance to common antibiotics in the challenge collection. MCM, mecillinam; TEM, temocillin; NTX, nitroxoline; FEP, cefepime; CTX, cefotaxime; CAZ, ceftazidime; TZP, piperacillin/tazobactam; MEM, meropenem.

Overall, meropenem [MIC_50/90_ <0.25/<0.25 mg/L, 2% resistant (R)] and nitroxoline (MIC_50/90_ 4/16 mg/L, 2% R) were the most active antibiotics *in vitro*, followed by mecillinam (MIC_50/90_ 2/128 mg/L, 15% R), temocillin (MIC_50/90_ 4/32 mg/L, 13% R) and cefepime (MIC_50/90_ 2/64 mg/L, 24% R) (Figure 1 and Table S1). MIC_50/90_ for other antibiotic agents was >64/>64 mg/L for cefotaxime (92% R), 16/>64 mg/L for ceftazidime (59% R), 8/>128 mg/L for piperacillin/tazobactam (41% R) and <0.5/1 mg/L for ertapenem (11% R).

In ESBL-positive isolates MIC_50/90_ was 4/32 mg/L (12% R) for mecillinam, 4/16 mg/L (7% R) for temocillin and 4/8 mg/L (1% R) for nitroxoline compared with 2/>128 mg/L (19% R) for mecillinam, 8/64 mg/L (27% R) for temocillin and 4/16 mg/L (2% R) for nitroxoline in AmpC isolates (Table 2). In carbapenemase-producing Enterobacterales (CPE) MICs of mecillinam and temocillin were higher (MIC_50/90_ 64/>128 mg/L for mecillinam, 67% R; MIC_50/90_ 128/>128 mg/L for temocillin, 75% R), while MICs of nitroxoline were similar to ESBL/AmpC-producing isolates (MIC_50/90_ 4/8 mg/L, 0% R) (Table 2).

### Mecillinam

Mecillinam showed excellent *in vitro* activity in *E. coli, Klebsiella aerogenes* and *Enterobacter* spp., despite ESBL and/or AmpC overexpression (Table S1). In CPE, susceptibility was limited to isolates with OXA-48-like carbapenemases and low carbapenem MICs (4/8, 50%) (Table S2).

Of note, among *Klebsiella* spp., 10/12 *K. aerogenes* isolates (83%) were susceptible to mecillinam compared with 50/66 *K. pneumoniae* isolates (76%) and 0/9 *K. oxytoca* isolates (0%).

Particularly poor activity was demonstrated for MDR *Proteus mirabilis* (MIC_50/90_ >128/>128 mg/L, 5/8 isolates R).

All three isolates of *Hafnia alvei* showed low mecillinam MICs (0.5–4 mg/L) despite derepressed AmpC, but so far no EUCAST breakpoint has been defined for this species.

### Temocillin

Stratified by species, temocillin was most active in *P. mirabilis*, *E. coli*, *K. oxytoca* and *K. pneumoniae* (Table 1). In ESBL-producing isolates temocillin was more active (20/273, 7% R) compared with mecillinam (34/273, 12% R), but less active compared with nitroxoline (4/273, 1% R).

As expected, no relevant activity was found in OXA-48-like producers while a KPC-expressing *Citrobacter freundii* and 2/3 MBL-producing isolates (VIM-1 and NDM-1, both *P. mirabilis*) were susceptible to temocillin (Table S2).

### Nitroxoline

Overall, nitroxoline demonstrated the highest *in vitro* activity in our challenge collection. In *E. coli* 99% (196/198) of isolates were nitroxoline susceptible. High nitroxoline MICs >16 mg/L were rare (*n *= 6) and were recorded in four ESBL-producing isolates [*E. coli* (*n *= 2), *K. pneumoniae* (*n *= 2)] and two isolates with overexpressed AmpC [*E. cloacae* (*n *= 2)]. The activity of nitroxoline was high irrespective of species and β-lactamase: MIC_50/90_ was 4/8 mg/L for isolates producing ESBLs, 4/16 mg/L for AmpC, 4/8 mg/L for CPE and 4/16 mg/L for isolates producing other β-lactamases.

### Comparison of methods for susceptibility testing

**Figure 2.**
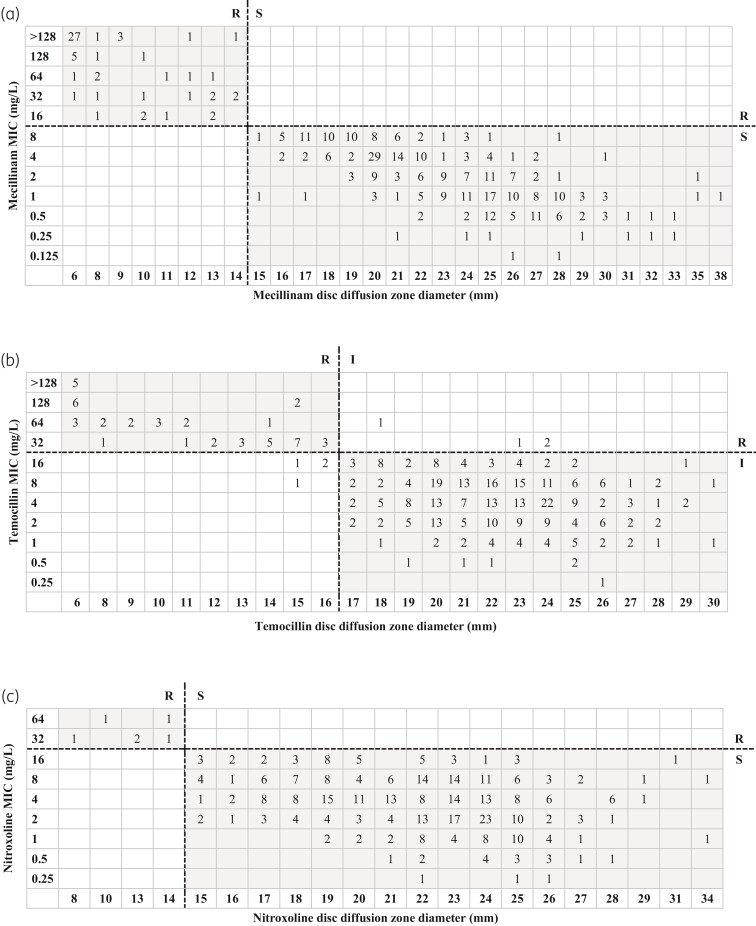
Comparison of MIC testing and disc diffusion for all isolates (*n *= 394). Dashed lines indicate EUCAST 12.0 breakpoints. S, susceptible at standard exposure; I, susceptible increased exposure; R, resistant. (a) Comparison of mecillinam agar dilution and disc diffusion. (b) Comparison of temocillin broth dilution and disc diffusion. (c) Comparison of nitroxoline broth dilution and disc diffusion.

The correlation of dilution methods and disc diffusion results over all species was excellent for mecillinam and moderate for temocillin and nitroxoline [Spearman’s correlation coefficient r = −0.837 for mecillinam, r = −0.474 for temocillin and r = −0.352 for nitroxoline (*P *< 0.01)] (Figure 2a–c and Figure S2a–c). For temocillin all errors concerned *Morganella morganii*, for which no EUCAST breakpoints have been defined.

## Discussion

This study compares the activities of three drugs in a collection of 394 MDR Enterobacterales isolates with different resistance mechanisms.

Compared with most other antibiotics, e.g. ceftazidime (59% R), piperacillin/tazobactam (41% R) or ciprofloxacin (54% R), the activity of mecillinam (15% R), temocillin (13% R) and nitroxoline (2% R) in this collection of MDR isolates was high.

Mecillinam was highly active against *E. coli, K. aerogenes* and *Enterobacter* spp. with ESBL production and/or AmpC overexpression, similar to results of previous studies.^14^ Susceptibility among CPE was limited to isolates with OXA-48-like carbapenemases with low carbapenem MICs, as previously shown.^15^ Some authors have suggested the use of mecillinam in cUTI or even bloodstream infections.^24,25^ However, only limited data on (piv)mecillinam serum and urinary levels are available.^26,27^ Higher maximum serum (peak 12 mg/L after 200 mg IV) and urinary concentrations have been shown after parenteral administration.^28^ Given the low mecillinam MICs recorded in the present study (198/394 isolates MIC ≤2 mg/L) the use of parenteral mecillinam could be a carbapenem-sparing alternative for infections with MDR Enterobacterales and should be further studied.

The activity of temocillin was high in *P. mirabilis*, *E. coli*, *K. oxytoca* and *K. pneumoniae* and in ESBL-producing isolates. In CPE, susceptibility was retained in isolates producing KPC, VIM-1 and NDM-1, but not in OXA-48-like producers as previously shown.^29^ However, these results have to be interpreted with caution as the number of CPE isolates in this study was low.

With the ongoing discussion on breakpoints and dosing it should be emphasized that the EUCAST temocillin breakpoints apply to high exposure (2 g q8h) for wild-type populations (MIC 1–16 mg/L).

Measured serum/urine concentrations for the above mentioned dosage scheme (peak 236 mg/L in serum and 68% in urine within 24 h) exceed MICs assessed in our study.^3,30^

In the present study Enterobacterales species without breakpoints (e.g. *E. cloacae* and *C. freundii*) were included and showed similar MICs compared with *E. coli* or *K. pneumoniae* (MIC_50/90_ 8/32 mg/L; 8/128 mg/L). Clinical success of temocillin treatment has been documented for infections caused by ESBL-producing isolates as well as Enterobacterales with derepressed AmpC.^10,31^ However, in line with other studies, in our cohort MICs were higher in presence of AmpC overexpression (MIC_50/90_ of 8/64 mg/L versus MIC_50/90_ of 4/16 mg/L in absence of AmpC).^31,32^

Overall our data demonstrate that temocillin has high activity in MDR Enterobacterales and may be used as an alternative drug to spare common broad spectrum antibiotics such as piperacillin/tazobactam or carbapenems.^32^ Temocillin could serve as a step-down therapy after susceptibility testing. However, more prospective data on the outcome of MDR infections treated with temocillin is needed, especially for those originating from non-urinary foci.

The highest susceptibility of Enterobacterales was observed for nitroxoline. Of particular interest, nitroxoline shows excellent activity in presence of carbapenemases and was more active in CPE than meropenem, as previously demonstrated.^12^

Data on serum and urine concentrations of nitroxoline are highly diverging (conjugated form: serum peak 5–9.5 mg/L; unconjugated form: serum peak 0.5–400 mg/L; conjugated form: urine peak 27–210 mg/L) and the role of the conjugated form is still unclear.^33,34^

However, serum and urine concentration levels of the unconjugated form exceed the MICs for most isolates of our collection, making nitroxoline a promising candidate for eradication of otherwise drug-resistant Enterobacterales in uUTI.

On the other hand, therapeutic failure has been reported for patients with UTI caused by *E. coli.*^35^ Thus, despite promising *in vitro* data more *in vivo* data are needed, especially on the correlation of microbiological success and clinical outcome of UTI treated with nitroxoline.

As MIC determination by agar dilution and broth microdilution is laborious and only few commercial assays for MIC determination are available, an alternative testing method is needed that can be carried out in clinical microbiology laboratories. Therefore, disc diffusion was assessed and results were compared with those from reference methods. Disc diffusion testing correlated excellently with MIC determination for mecillinam (r = −0.837), while for temocillin (r = −0.474) and nitroxoline (r = −0.352) the correlation was lower. It has to be taken into account that the number of resistant isolates was low for all three drugs, which limits the assessment of the correlation between MIC and inhibition zones.

No major or very major errors were recorded for mecillinam disc testing, which have previously been described for CPE isolates. This indicates that overestimation of mecillinam susceptibility in MDR Enterobacterales might be limited to some CPE, but does not apply to isolates with other resistance mechanisms.^15^

Overall, in this cohort of MDR Enterobacterales, therapeutical options are limited and mecillinam, temocillin and nitroxoline are valuable assets for UTI treatment. Isolates resistant to nitroxoline were very rare, despite expression of ESBL, AmpC overexpression or even carbapenemases. Our data further demonstrate that mecillinam and temocillin are often hydrolysed to a lesser extent than other β-lactams.^36^ In MDR Enterobacterales mecillinam may be particularly promising for ESBL-, AmpC- or OXA-48-like-producing *E. coli* and *Enterobacter* spp., while temocillin is particularly active in ESBL-expressing isolates.

Our study has some limitations. Most samples were from inpatients and therefore might not be completely representative for uUTI. The strength of our study is that it includes many MDR isolates compared with previous studies, and additionally assessed species other than *E. coli* including those without currently defined breakpoints. Additionally, we provide data on the performance of disc diffusion compared with MIC determination. This will likely be helpful for the determination of susceptibility in MDR isolates in routine laboratories that cannot perform susceptibility testing with laborious reference methods.

With the problem of continuously increasing AMR, all three drugs should be further investigated with *in vivo* studies either as definite therapy or as part of a combination therapy for MDR Enterobacterales.

## Supplementary Material

dlac059_Supplementary_Data

## Supplementary data

Tables S1 and S2 and Figures S1 and S2 are available as Supplementary data at *JAC-AMR* Online.
