# Supplementary material for: In vitro activity of mecillinam, temocillin and nitroxoline against MDR Enterobacterales
Source: JAC Antimicrob Resist. 2022 Jun 16;4(3):dlac059. doi: 10.1093/jacamr/dlac059 (PMC9201239; doi:10.1093/jacamr/dlac059)
Supplement: dlac059_Supplementary_Data [file dlac059_supplementary_data.docx]

**Supplementary data**


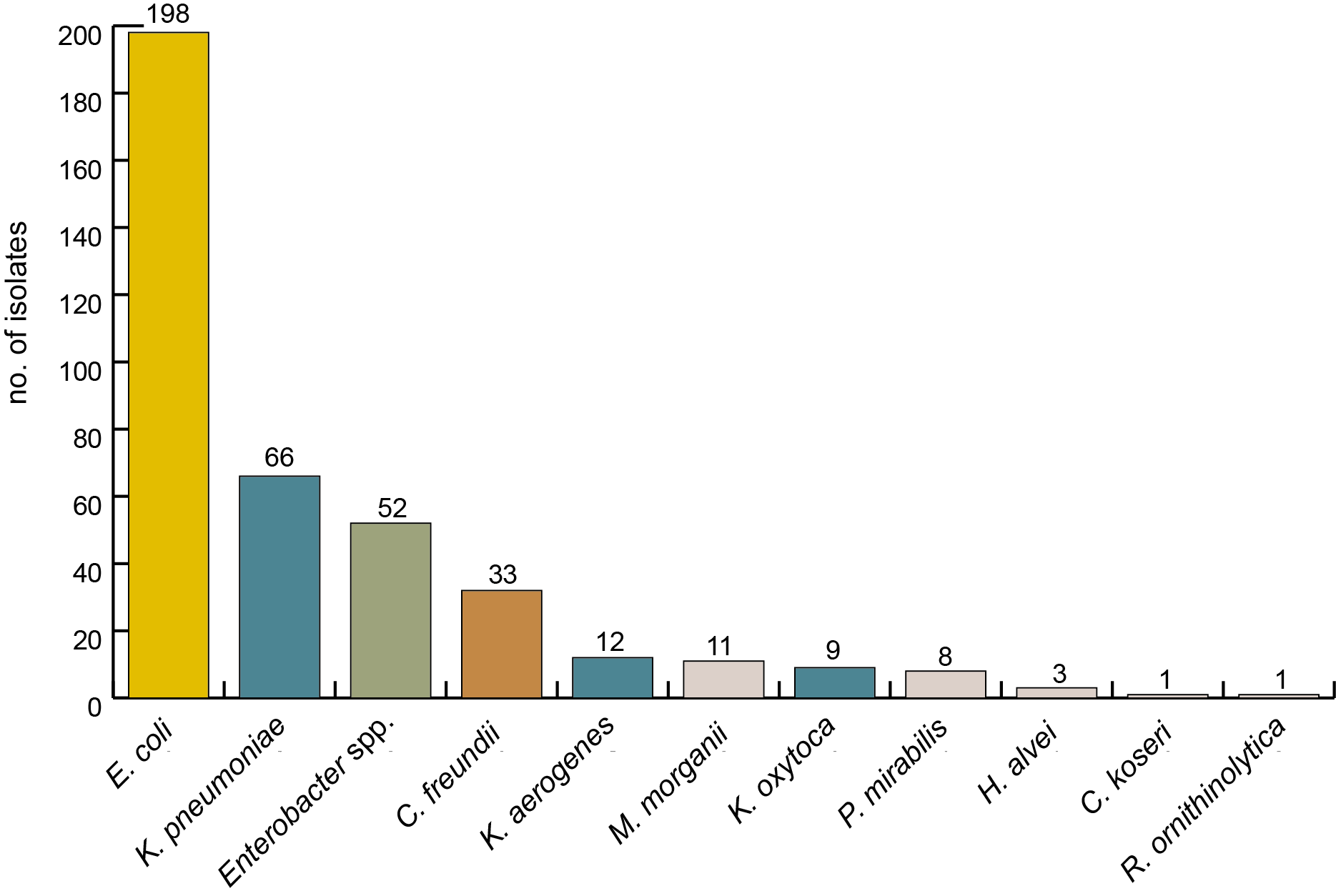


**species**

**Figure S1.** Species distribution of isolates included in the study.

**Table S1.** Minimal inhibitory concentrations of isolates to mecillinam, temocillin, nitroxoline and comparators

| **Species (number)** | **Mecillinam** | | | **Temocillin** | | | **Nitroxoline** | | | **Piperacillin/tazobactam** | | | **Meropenem** | | | |
| --- | --- | --- | --- | --- | --- | --- | --- | --- | --- | --- | --- | --- | --- | --- | --- | --- |
|  | **MIC_50_ (mg/L)** | **MIC range (mg/L)** | **Resistant isolates** | **MIC_50_ (mg/L)** | **MIC range (mg/L)** | **Resistant isolates** | **MIC_50_ (mg/L)** | **MIC range (mg/L)** | **Resistant isolates** | **MIC_50_ (mg/L)** | **MIC range (mg/L)** | **Resistant isolates** | **MIC_50_  (mg/L)** | **MIC range (mg/L)** | **Resistant isolates** |  |
| ***E. coli (*198)** | **2** | **0.25 – 64** | **6 (3%)** | **4** | **0.5 – >128** | **8 (4%)** | **2** | **0.25 – 32** | **2 (1%)** | **<4** | **<4 – >128** | **34 (17%)** | **<0.25** | **<0.25 – 1** | **0** |  |
| ESBL (177) | 2 | 0.25 – 64 | 5 (3%) | 4 | 0.5 – 128 | 4 (2%) | 2 | 0.25 – 32 | 2 (1%) | <4 | <4 – >128 | 22 (12%) | <0.25 | <0.25 | 0 |  |
| AmpC (11) | 1 | 0.5 – 4 | 0 | 8 | 2 – 16 | 0 | 4 | 0.5 – 8 | 0 | 16 | <4 – >128 | 6 (55%) | <0.25 | <0.25 | 0 |  |
| ESBL + AmpC (7) | 4 | 1 – 8 | 0 | 4 | 1 – 64 | 1 (14%) | 4 | 2 – 16 | 0 | <4 | <4 – >128 | 3 (43%) | <0.25 | <0.25 | 0 |  |
| OXA-181 (1) |  | 1 | 0 |  | >128 | 1 (100%) |  | 8 | 0 |  | >128 | 1 (100%) |  | 1 | 0 |  |
| OXA-181+ ESBL (1) |  | 4 | 0 |  | 64 | 1 (100%) |  | 2 | 0 |  | >128 | 1 (100%) |  | 1 | 0 |  |
| OXA-244 (1) |  | 32 | 1 (100%) |  | 64 | 1 (100%) |  | 8 | 0 |  | >128 | 1 (100%) |  | 0.5 | 0 |  |
| ***Klebsiella* spp. (87)** | **8** | **0.5 – >128** | **27 (31%)** | **4** | **0.5 – >128** | **13 (15%)** | **4** | **1 – 64** | **2 (2%)** | **16** | **<4 – >128** | **52 (60%)** | **<0.25** | **<0.25 – >16** | **3 (3%)** |  |
| ***K. pneumoniae* (66)** | **4** | **0.5 – >128** | **16 (24%)** | **4** | **0.5 – >128** | **8 (12%)** | **4** | **1 – 64** | **2 (3%)** | **8** | **<4 – >128** | **31 (47%)** | **<0.25** | **<0.25 – >16** | **3 (5%)** |  |
| ESBL (60) | 4 | 0.5 – >128 | 12 (20%) | 4 | 0.5 – >128 | 4 (7%) | 4 | 1 – 64 | 2 (3%) | 8 | <4 – >128 | 26 (43%) | <0.25 | <0.25 | 0 |  |
| AmpC (1) |  | 2 | 0 |  | 4 | 0 |  | 8 | 0 |  | 8 | 0 |  | <0.25 | 0 |  |
| ESBL + AmpC (1) |  | 4 | 0 |  | 64 | 1 (100%) |  | 8 | 0 |  | >128 | 1 (100%) |  | <0.25 | 0 |  |
| SHV (1) |  | >128 | 1 (100%) |  | 8 | 0 |  | 16 | 0 |  | >128 | 1 (100%) |  | 4 | 0 |  |
| OXA-232 + ESBL (3) | 64 | 64 – 128 | 3 (100%) | 128 | ≥128 | 3 (100%) | 2 | 1 – 4 | 0 | >128 | >128 | 3 (100%) | >16 | >16 | 3 (100%) |  |
| ***K. aerogenes (*12)** | **2** | **1 – >128** | **2 (17%)** | **8** | **2 – 128** | **4 (33%)** | **4** | **1 – 8** | **0** | **>128** | **16 – >128** | **12 (100%)** | **<0.25** | **<0.25 – 0.5** | **0** |  |
| AmpC (11) | 2 | 1 – >128 | 1 (9%) | 8 | 2 – 128 | 3 (27%) | 4 | 1 – 8 | 0 | >128 | 16 – >128 | 11 (100%) | 0.5 | <0.25 **–** 0.5 | 0 |  |
| ESBL + AmpC (1) |  | >128 | 1 (100%) |  | 64 | 1 (100%) |  | 8 | 0 |  | >128 | 1 (100%) |  | <0.25 | 0 |  |
| ***K. oxytoca* (n=9)** | **64** | **32 – >128** | **9 (100%)** | **4** | **1 – 32** | **1 (11%)** | **4** | **1 – 8** | **0** | **16** | **16 – >128** | **9 (100%)** | **<0.25** | **<0.25** | **0** |  |
| ESBL (8) | 32 | 32 – >128 | 8 (100%) | 4 | 1 – 32 | 1 (13%) | 4 | 1 – 8 | 0 | 16 | 16 – >128 | 8 (100%) | <0.25 | <0.25 | 0 |  |
| HyperK1 (1) |  | >128 | 1 (100%) |  | 8 | 0 |  | 4 | 0 |  | >128 | 1 (100%) |  | <0.25 | 0 |  |
| ***Enterobacter* spp. (52)** | **1** | **0.125– >128** | **2 (4%)** | **8** | **0.5 – 128** | **16 (31%)** | **8** | **0.5 – 64** | **2 (4%)** | **>128** | **<4 – >128** | **41 (79%)** | **<0.25** | **<0.25 – 2** | **0** |  |
| ESBL (1) |  | 4 | 0 |  | 32 | 1 (100%) |  | 16 | 0 |  | 16 | 1 (100%) |  | <0.25 | 0 |  |
| AmpC (50) | 1 | 0.125– >128 | 2 (4%) | 8 | 0.5 – 128 | 15 (30%) | 8 | 0.5 – 64 | 2 (4%) | >128 | <4 – >128 | 40 (80%) | <0.25 | <0.25 – 2 | 0 |  |
| ESBL + AmpC (1) |  | 1 | 0 |  | 1 | 0 |  | 2 | 0 |  | <4 | 0 |  | <0.25 | 0 |  |

| **Species (number)** | **Mecillinam** | | | **Temocillin** | | | **Nitroxoline** | | | **Piperacillin/tazobactam** | | | **Meropenem** | | | |
| --- | --- | --- | --- | --- | --- | --- | --- | --- | --- | --- | --- | --- | --- | --- | --- | --- |
|  | **MIC_50_ (mg/L)** | **MIC range (mg/L)** | **Resistant isolates** | **MIC_50_ (mg/L)** | **MIC range (mg/L)** | **Resistant isolates** | **MIC_50_ (mg/L)** | **MIC range (mg/L)** | **Resistant isolates** | **MIC_50_ (mg/L)** | **MIC range (mg/L)** | **Resistant isolates** | **MIC_50_  (mg/L)** | **MIC range (mg/L)** | **Resistant isolates** |  |
| ***C. freundii* (33)** | **2** | **0.125– >128** | **9 (27%)** | **8** | **0.25 – >128** | **11 (33%)** | **4** | **0.5 – 16** | **0** | **64** | **<4 – >128** | **27 (82%)** | **<0.25** | **<0.25 – >16** | **2 (6%)** |  |
| ESBL (1) |  | 8 | 0 |  | 8 | 0 |  | 16 | 0 |  | 32 | 1 (100%) |  | <0.25 | 0 |  |
| AmpC (24) | 1 | 0.125–>128 | 4 (17%) | 8 | 0.25– >128 | 6 (25%) | 4 | 0.5 – 16 | 0 | 32 | <4 – >128 | 18 (75%) | <0.25 | <0.25 – 4 | 0 |  |
| ESBL + AmpC (4) | 16 | 4 – >128 | 3 (75%) | 8 | 4 – 64 | 2 (50%) | 8 | 4 – 16 | 0 | >128 | >128 | 4 (100%) | <0.25 | <0.25 | 0 |  |
| KPC-3 + AmpC (1) |  | >128 | 1 (100%) |  | 4 | 0 |  | 0.5 | 0 |  | >128 | 1 (100%) |  | >16 | 1 (100%) |  |
| OXA-48 + AmpC (1) |  | 4 | 0 |  | 128 | 1 (100%) |  | 2 | 0 |  | >128 | 1 (100%) |  | 1 | 0 |  |
| OXA-48+ESBL/AmpC (1) |  | 8 | 0 |  | 128 | 1 (100%) |  | 4 | 0 |  | >128 | 1 (100%) |  | 2 | 0 |  |
| VIM-1 + AmpC (1) |  | >128 | 1 (100%) |  | 128 | 1 (100%) |  | 4 | 0 |  | >128 | 1 (100%) |  | >16 | 0 |  |
| ***C. koseri* (1)** |  | **8** | **0** |  | **2** | **0** |  | **4** | **0** |  | **<4** | **0** |  | **<0.25** | **0** |  |
| ESBL (1) |  | 8 | 0 |  | 2 | 0 |  | 4 | 0 |  | <4 | 0 |  | <0.25 | 0 |  |
| ***M. morganii* (11)** | **>128** | **128 – >128** | **11 (100%)** | **16** | **4 – 64** | **4 (36%)** | **4** | **0.5 – 16** | **0** | **<4** | **<4 – >128** | **2 (18%)** | **<0.25** | **<0.25 – 0.5** | **0** |  |
| ESBL (1) |  | >128 | 1 (100%) |  | 8 | 0 |  | 16 | 0 |  | <4 | 0 |  | <0.25 | 0 |  |
| AmpC (10) | >128 | ≥128 | 10 (100%) | 16 | 4 – 64 | 4 (40%) | 4 | 0.5 – 8 | 0 | <4 | <4 – >128 | 2 (20%) | <0.25 | <0.25 – 0.5 | 0 |  |
| ***P. mirabilis* (8)** | **>128** | **2 – >128** | **5 (63%)** | **2** | **1 – 4** | **0** | **4** | **1 – 8** | **0** | **<4** | **<4 – >128** | **2 (25%)** | **<0.25** | **<0.25 – >16** | **2 (25%)** |  |
| ESBL (3) | 4 | 2 – >128 | 1 (33%) | 2 | 1 – 4 | 0 | 4 | 4 – 8 | 0 | <4 | <4 | 0 | <0.25 | <0.25 | 0 |  |
| AmpC (2) | >128 | >128 | 2 (100%) | 2 | 2 – 4 | 0 | 1 | 1 – 1 | 0 | <4 | <4 | 0 | <0.25 | <0.25 | 0 |  |
| ESBL + AmpC (1) |  | 8 | 0 |  | 2 | 0 |  | 8 | 0 |  | <4 | 0 |  | <0.25 | 0 |  |
| NDM-1 (1) |  | >128 | 1 (100%) |  | 2 | 0 |  | 8 | 0 |  | >128 | 1 (100%) |  | >16 | 1 (100%) |  |
| VIM-1 (1) |  | >128 | 1 (100%) |  | 4 | 0 |  | 4 | 0 |  | >128 | 1 (100%) |  | >16 | 1 (100%) |  |
| ***H. alvei* (3)** | **2** | **0.5 – 4** | **0** | **8** | **4 – 8** | **0** | **2** | **2** | **0** | **16** | **8 – >128** | **1 (33%)** | **<0.25** | **<0.25** | **0** |  |
| AmpC (3) | 2 | 0.5 – 4 | 0 | 8 | 4 – 8 | 0 | 2 | 2 | 0 | 16 | 8 – >128 | 1 (33%) | <0.25 | <0.25 | 0 |  |
| ***R. ornithinolytica* (1)** |  | **2** | **0** |  | **8** | **0** |  | **8** | **0** |  | **16** | **1 (100%)** |  | **<0.25** | **0** |  |
| ESBL + AmpC (1) |  | 2 | 0 |  | 8 | 0 |  | 8 | 0 |  | 16 | 1 (100%) |  | <0.25 | **0** |  |
| **All isolates (394)** | **2** | **0.125– >128** | **60 (15%)** | **4** | **0.25– >128** | **52 (13%)** | **4** | **0.25 – 64** | **6 (2%)** | **8** | **<4 – >128** | **161 (41%)** | **<0.25** | **<0.25 – >16** | **7 (2%)** |  |

**Table S2.** Results of MIC and disk diffusion testing for carbapenemase producing isolates; S=susceptible at standard dose; I=susceptible increased exposure; R=resistant

|  |  | **Mecillinam** | | **Temocillin** | | **Nitroxoline** | |
| --- | --- | --- | --- | --- | --- | --- | --- |
| **β-lactamase content** | **Species** | **MIC**  **(mg/L)** | **DD (mm)** | **MIC**  **(mg/L)** | **DD (mm)** | **MIC**  **(mg/L)** | **DD (mm)** |
| **KPC-3, CMY-65** | *C. freundii* | >128 [R] | 6 | 4 [I] | 22 | 0.5 [S] | 22 |
| **NDM-1** | *P. mirabilis* | >128 [R] | 6 | 2 [I] | 23 | 8 [S] | 21 |
| **OXA-48, CMY-48** | *C. freundii* | 4 [S] | 20 | 128 [R] | 6 | 2 [S] | 22 |
| **OXA-48, CTX-M-9, CTX-M-3, CMY-152** | *C. freundii* | 8 [S] | 21 | 128 [R] | 6 | 4 [S] | 23 |
| **OXA-181** | *E. coli* | 1 [S] | 23 | >128 [R] | 6 | 8 [S] | 22 |
| **OXA-181, CTX-M-15** | *E. coli* | 4 [S] | 20 | 64 [R] | 6 | 2 [S] | 24 |
| **OXA-232, CTX-M-15** | *K. pneumoniae* | 64 [R] | 11 | 128 [R] | 6 | 1 [S] | 22 |
| **OXA-232, CTX-M-15** | *K. pneumoniae* | 64 [R] | 8 | >128 [R] | 6 | 2 [S] | 22 |
| **OXA-232, CTX-M-15** | *K. pneumoniae* | 128 [R] | 6 | >128 [R] | 6 | 4 [S] | 23 |
| **OXA-244** | *E. coli* | 32 [R] | 14 | 64 [R] | 8 | 8 [S] | 23 |
| **VIM-1, CMY-78** | *C. freundii* | >128 [R] | 6 | 128 [R] | 6 | 4 [S] | 21 |
| **VIM-1** | *P. mirabilis* | 128 [R] | 6 | 4 [I] | 25 | 4 [S] | 25 |


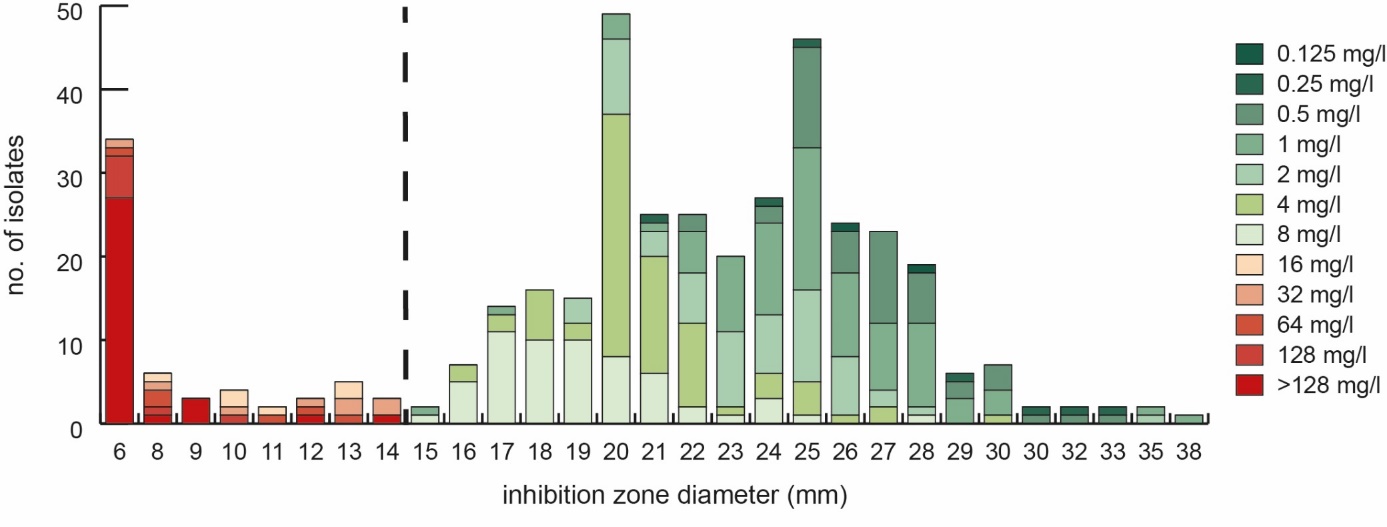


mecillinam

(a)

(c)

(b)

temocillin


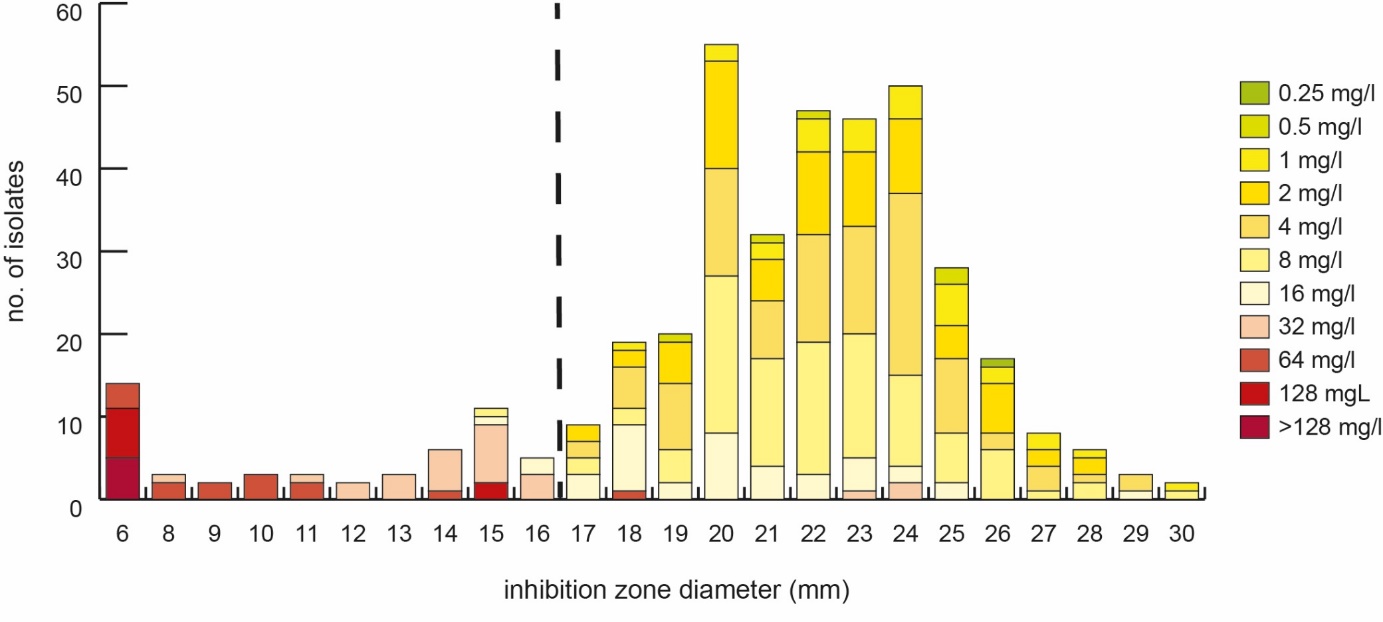


nitroxoline


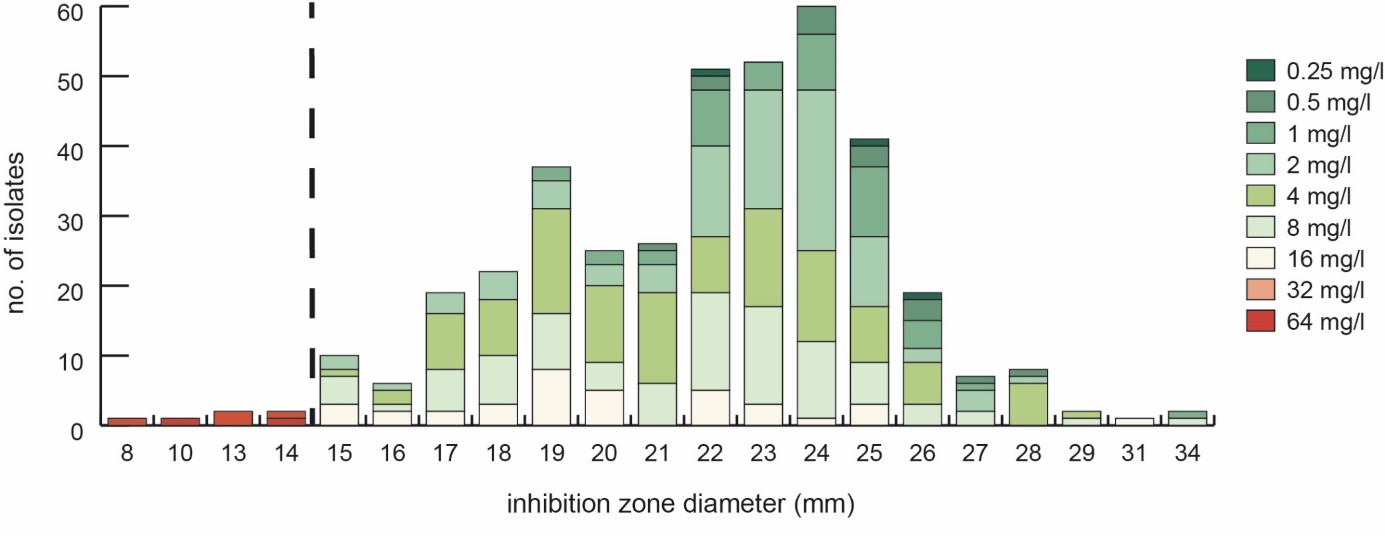


**Figure S2.** MICs of clinical isolates versus respective inhibition zone by disk diffusion. Dashed line indicates EUCAST Breakpoint v12.0; (a) mecillinam; (b) temocillin; (c) nitroxoline.
